# Differential gene methylation and expression of *HOX* transcription factor family in orbitofacial neurofibroma

**DOI:** 10.1186/s40478-020-00940-7

**Published:** 2020-05-04

**Authors:** Antje Arnold, Eddie Luidy Imada, Lisa Zhang, Deepak P. Edward, Luigi Marchionni, Fausto J. Rodriguez

**Affiliations:** 1 Departments of Pathology, Johns Hopkins University School of Medicine, Baltimore, MD United States; 2 Departments of Ophthalmology and Sidney Kimmel Comprehensive Cancer Center, Johns Hopkins University School of Medicine, Baltimore, Maryland (MD) USA; 3 Department of Pathology, Massachusetts General Hospital, Boston, MA USA; 4 King Khaled Eye Specialist Hospital, Riyadh, Saudi Arabia; 5 Department of Ophthalmology and Visual Sciences, University of Illinois College of Medicine, Chicago, IL USA; 6 Johns Hopkins University School of Medicine, Sheikh Zayed Tower, Room M2101, 1800 Orleans Street, Baltimore, MD 21231 USA

**Keywords:** Neurofibromatosis, Orbitofacial, Neurofibromas, Plexiform neurofibromas, HOX genes

## Abstract

Although most commonly benign, neurofibromas (NFs) can have devastating functional and cosmetic effects in addition to the possibility of malignant transformation. In orbitofacial neurofibromatosis type 1, NFs may cause progressive, disfiguring tumors of the lid, brow, temple, face and orbit. The purpose of this study was to identify biological differences between orbitofacial NFs and those occurring at other anatomic sites. We used Illumina Methylation EPIC BeadChip to study DNA methylation differences between orbitofacial NFs (*N* = 20) and NFs at other sites (*N* = 4). Global methylation differences were detected between the two groups and the top differentially methylated genes were part of the *HOX (Homebox)* family of transcription factors (*HOXC8, HOXC4, HOXC6, HOXA6 and HOXD4*), which were hypomethylated in orbitofacial NFs compared to the non-orbital NFs. Conversely, *LTF* (lactoferrin) was relatively hypermethylated in orbitofacial NF compared to non-orbitofacial NF. HOXC8 protein levels were higher in orbitofacial plexiform NFs (*p* = 0.04). We found no significant differences in the expression of *HOXC4, HOXA6, or HOXD4* between the two groups. *HOXC8* mRNA levels were also higher in orbitofacial NFs and *HOXC8* overexpression in a non-neoplastic human Schwann cell line resulted in increased growth. In summary, we identified gene methylation and expression differences between orbitofacial NF and NFs occurring at other locations. Further investigation may be warranted, given that the *HOX* family of genes play an important role during development, are dysregulated in a variety of cancers, and may provide novel insights into therapeutic approaches.

## Introduction

Neurofibromatosis type 1 (NF1), also known as von Recklinghausen disease, is a relatively frequent (1 in 3000 live births), autosomal dominant, neurocutaneous disorder with heterogeneous clinicopathologic manifestations [38]. The most prevalent tumor in NF1 patients is neurofibroma (NF), which is typically slow growing and benign, but can have devastating functional and cosmetic effects. Malignant transformation develops in 5–10% of NF1 patients, typically in a plexiform neurofibromas subtype [45].

**Fig. 1:**
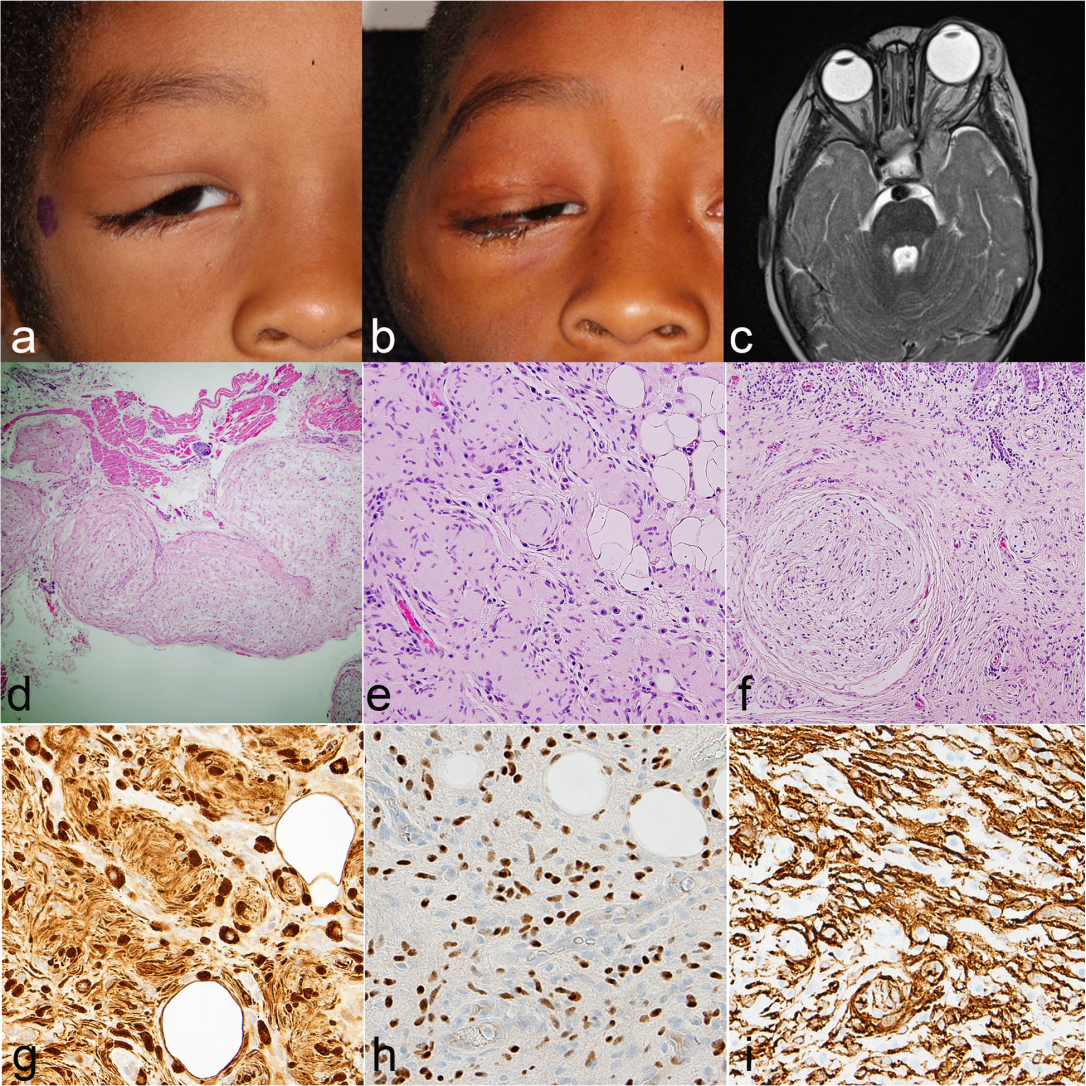
Clinicopathologic features of orbitofacial neurofibroma. Five year-old boy with neurofibromatosis type 1 with orbitofacial neurofibroma at first presentation (**a**) and recurrence 3 years post-treatment (**b**). Neurofibromas in this region are notoriously difficult to treat and may involve a variety of orbitofacial structures. This example extends to the ipsilateral cavernous sinus (**c**). Histologic evaluation in these tumors frequently demonstrates plexiform (**d**) and diffuse (**e**) patterns, frequently coexisting (**f**). Immunohistochemistry consistently demonstrates strong expression of S100 (**g**), SOX10 (**h**), and CD34 (**i**)

Orbitofacial neurofibromatosis type 1 (OFNF) has been recognized as a unique clinical variant of NF1 for many years [6]. In OFNF, which occurs in 1–22% of patients, orbitofacial NFs may cause progressive, disfiguring tumors of the lid, brow, temple, face and orbit [11, 33, 48]. Large NFs involving the orbit tend to be more aggressive and infiltrative compared to NFs located elsewhere in the body (Fig. 1) [18]. The recurrence rate after excision is also high. In addition, this aggressiveness is most striking in infancy and early childhood and tends to improve somewhat as the individual ages [18, 23, 24].

These tumors not only affect facial appearance [37], but they can also disturb the growth patterns of the developing skull [19, 49], orbit [20, 43], and globe [15, 30]. Sphenoid and orbital dysplasia are prominent. Skull and facial features ipsilateral to neurofibromas include enlargement of the middle cranial fossa and defects in the greater sphenoid wing and lateral orbital wall. In addition, globe enlargement occurs on the affected side of patients with OFNF under certain circumstances [15, 30]. Both neuroimaging [19, 20, 49] and clinical reports [29] have now documented that NFs are frequently contiguous to globes that are abnormally large and to bony changes in the skull and orbit [13]. However, the reasons that orbitofacial NFs are more aggressive than NFs at other locations, and the mechanisms by which they disturb developing skull and facial structures are currently unclear.

Orbitofacial NFs designation is usually applied to patients with often complex NFs that, given the infiltrative nature, involve the orbit and ocular adnexa, but also frequently adjacent facial structures. We previously reviewed our pathology experience with nerve sheath tumors involving the eye and ocular adnexa. Neurofibroma was the predominant nerve sheath tumor type involving this region. Of note, 63 neurofibromas at this location were studied, and they were predominantly plexiform, diffuse or with mixed patterns (68%) and 89% develop in patients with known NF1 [48]. Although orbitofacial NFs often behave in an aggressive fashion, malignant transformation appears to be paradoxically rare. We only encountered a single case of malignant peripheral nerve sheath tumor (MPNST) in our review of 90 nerve sheath tumors from 67 patients involving the eye and ocular adnexa [48]. This patient was a 63-year-old woman who underwent clinical progression of a childhood orbitofacial neurofibroma over several decades, with eventual malignant transformation, dissemination and death [39].

Recent advances in our understanding of human neoplasia have uncovered an important role for epigenetic changes in addition to DNA-level mutations and copy number changes. Furthermore, methylation profiling has emerged as a robust tool to aid in the classification of tumors of the nervous system [5], including those of peripheral nerve origin [40]. DNA methylation profiles vary according to tumor subtypes. For example, MPNST, an aggressive malignant neoplasm that can develop in NF1 patients, is characterized by relative global hypomethylation [32]. Another rationale to study epigenetic changes in subsets of NFs is that genetic alterations in these slow-growing tumors are essentially limited to *NF1* mutations [35]. We hypothesized that global methylation profiling would be helpful in studying biological differences between orbitofacial NFs and those developing in other body sites.

## Materials and methods

### Patients and samples

Formalin fixed, paraffin embedded tissues of 85 neurofibromas from 63 patients were studied by methylation array, qPCR and/or immunohistochemistry as described below. Only larger tumors with adequate material for DNA, RNA, and/or IHC analysis were selected, which excluded small localized tumors and enriched for larger more clinically significant tumors (more properly designated orbitofacial NF). Demographics and basic location characteristics were abstracted from retrospective review of clinical and pathologic records. Orbitofacial (*n* = 34) and non-orbitofacial (*n* = 51) tumors were studied. Pathologic subtypes included plexiform (*n* = 43), diffuse (*n* = 15), localized intraneural (n = 4) and localized cutaneous (*n* = 23). Clinicopathologic characteristics and tests performed per tumor are summarized in Supplementary table 1.

### DNA methylation profiling

Formalin-fixed paraffin-embedded (FFPE) tissue from 20 orbitofacial and 4 non-orbital NFs were included for methylation profiling studies. In brief, FFPE sections were used to isolate genomic DNA with the QIAamp DNA FFPE Tissue Kit (Qiagen) and stored at 4 °C. Genomic DNA quality was assessed by low concentration agarose gel (0.6%) electrophoresis and fluorescent spectrometry with the PicoGreen DNA Kit (Life Technologies).

DNA bisulfite conversion was carried out using the EZ DNA Methylation Kit (Zymo Research) per manufacturer instructions with modifications for the Illumina Infinium Methylation Assay. Briefly, 400 ng of genomic DNA was first mixed with 5 μl of M-Dilution Buffer and incubated at 37 °C for 15 min, then mixed with 100 μl of CT Conversion Reagent prepared per the instruction manual. Mixtures were incubated in a thermocycler with 16 thermal cycles at 95 °C for 30 s and 50 °C for 1 h. Bisulfite-converted DNA samples were loaded onto 96-column plates provided in the kit for desulphonation and purification. Concentration of eluted DNA was measured using the Nanodrop-1000 spectrometer.

Bisulfite-converted DNA was analyzed using Illumina’s Infinium Human MethylationEPIC Beadchip Kit (WG-317-1002) per manufacturer instructions. The Beadchip contains over 850,000 CpG loci in human genome. Briefly, 4 μl of bisulfite-converted DNA was added to a 0.8 ml 96-well storage plate (Thermo Scientific), denatured in 0.014 N sodium hydroxide, and neutralized and amplified with kit-provided reagents and buffer at 37 °C for 20–24 h. Samples were fragmented using kit-provided reagents and buffer at 37 °C for 1 h and precipitated by adding 2-propanol. Re-suspended samples were denatured in a 96-well plate heat block at 95 °C for 20 min. 26 μl of each sample was loaded onto an 8-sample chip and the chips were assembled into hybridization chamber per manufacturer instructions. After incubation at 48 °C for 16–20 h, chips were briefly washed and then assembled and placed in a fluid flow-through station for primer-extension and staining procedures. Polymer-coated chips were image-processed in Illumina’s iScan scanner.

### Array data acquisition and bioinformatic analysis

The quality of the raw data was assessed in an R environment. Briefly, the raw data was inputted and processed using the *minfi* R/Bioconductor [2] package to obtain the Beta- and M-values (log ratio of beta). Standard quality controls were performed (e.g. density plots and control probes expression profiles). Probes within 300 base pairs of each other were analyzed as a single cluster. Differentially methylated regions (DMRs) were detected by fitting a linear model with age as a covariate to adjust for differences between pediatric and adult patients. For each cluster using the bumphunter R/Bioconductor package [21] to compute the ﻿family-wide error rates (FWER) and *p*-values based on 500 bootstraps to estimate a null-profile. The top 5% regions with FWER ≤ 0.1 were selected as candidates for validation. Each DMR was annotated to its nearest gene based on the hg19 assembly.

### RNA isolation and quantitative real time PCR analysis

The RNA was extracted using the RNeasy FFPE Kit (Qiagen). RNA concentration and purity were assessed using NanoDrop equipment (NanoDrop Technologies Inc.). One microgram of total RNA was used to produce cDNA with the super script III kit (INVITROGEN) and quantitative PCR was performed using SYBR Green reagents (Applied Biosystems, California) on an I-Cycler IQ Real-Time detection system (Bio-Rad, California) per manufacturer instructions. Primer sequences were as followed: huHOXC8 primers 5′-CTGTAAATCCTCCGCCAACAC-3′ (forward), 5′-CTTCAATCCGACGTTTTCGTG-3′ (reverse); huHOXC4 primers 5′-CCAGCAAGCAACCCATAGTC-3′ (forward), 5′-ATCCTTCTCCTTCGGGTCAG-3′ (reverse); huHOXA6 5′- CAAAGCACTCCATGACGAAGG-3′ (forward), 5′- CTCCTTCTCCAGCTCCAGTGT-3′ (reverse); GAPDH primers 5′- GCA GGG GGG AGC CAA AAG GGT-3′ (forward), 5′- TGG GTG GCA GTG ATG GCA TGG-3′ (reverse). The ΔΔ*C*_t_ method for quantitation of relative gene expression was used to determine the mean expression of each target gene normalized to the geometric mean of β-actin and GAPDH.

### Tissue microarray construction and immunohistochemistry

A total of 77 NFs, including 44 orbitofacial and 31 non-periorbital, were examined by immunohistochemistry. Clinicopathologic features of the orbitofacial tumors were reported in a prior study [48]. Tissue microarrays were constructed using four cores (0.6 mm diameter) from FFPE blocks. FFPE sections were deparaffinized in xylene, and antigen retrieval was performed thrice using sodium citrate buffer (pH 6.0) in a microwave oven at 5 min each. Sections were stained with rabbit polyclonal anti-HOXC4 (Abcam; 1:50 dilution, overnight at 4 °C), rabbit polyclonal anti-HOXC8 (Abcam; 1:50 dilution, overnight at 4 °C), rabbit polyclonal anti-HOXD4 (Novus Biologicals; 1:200, overnight at 4 °C); rabbit polyclonal anti-HOXA6 (Novus Biologicals; 1:200, overnight at 4 °C), followed by an anti-rabbit IgG secondary antibody staining with the VECTASTAIN® Elite® ABC HRP Kit (vectorlabs). After a diaminobenzidine reaction (Liquid DAB + Substrate Chromogen System, DakoCytomation), sections were counterstained with hematoxylin. All H&E slides were reviewed by one author (FJR), who scored the blinded samples. Immunoreactivity was scored semi-quantitatively based on the intensity of staining (0, no; 1, weak; 2, moderate; 3, intense). An overall immunohistochemical H-score (intensity x percentage of positive tumor cells) was assigned as low (H-score 0–100), medium (100–200), or high (200–300), as previously described [32]. Proliferation index was evaluated as a visual estimate of the percentage of positive neoplastic nuclei using a Ki67 antibody (Ventana, clone 30–9).

### Cell culture and transfection

A human Schwann cell line was obtained from *ScienCell* (Sciencell research laboratories) and maintained in complete Schwann Cell Medium (Sciencell research laboratories). All cell cultures were maintained at 37 °C in a humidified 5% CO_2_ atmosphere.

### DNA constructs and viral infection

The construct encoding *HOXC8* was purchased from Addgene (Addgene plasmid 21,001). The *HOXC8* cDNA was subcloned into the retroviral vector pBABE_puro. Retroviral particles were produced by transfecting 293GT cells with VSVG envelope plasmid and the plasmid of interest, using Lipofectamine2000 (ThermoFisher) per manufacturer instructions. After 24 h, the medium was changed to human Schwann cell media, and supernatants were collected at 48 and 72 h. These supernatants were pooled, passed through a 0.45-mm filter, and then stored at − 80 °C until further needed. Human Schwann cells were incubated with retroviral particles in combination with polybrene for 24 h. After approximately 1 week in culture, puromycin (ThermoFisher) at 2 μg/ml was added to the cell culture medium to select for stable *HOXC8*-overexpressing cells. Control cells were infected with empty vector viral particles.

### CellTiter-blue cell viability assay

Human Schwann cell line and human Schwann cell line transduced with *HOXC8* were seeded in a 96-well plate (1000 cell/well). On the following day (day 0), day 4, and day 6, 40 μl of Cell titer Blue Reagent (Promega) was added to 200 μl culture medium for 4 h at 37 °C; fluorescence with excitation 560 nm and emission 590 nm was measured using the TECAN reader (TECAN).

### Western blot analysis

Protein extraction, separation, and immunoblotting were performed as previously described [31]. All Western blots were representative of three independent experiments. Antibodies used were as follows: rabbit polyclonal anti-HOXC8 (Abcam; 1:1000), β-ACTIN (Santa Cruz; 1:5000), and GAPDH (Santa Cruz; 1:5000). Anti-mouse IgG HRP-linked (#7076S) and anti-rabbit IgG HRP-linked (#7074S) (Cell Signaling Technology) were used as secondary antibodies. Overexpression lysates for recombinant human HOXC8 were used as positive controls (Origene; NM_022658).

## Results

### HOX family of transcription factors are hypomethylated in orbitofacial neurofibromas

**Fig. 2:**
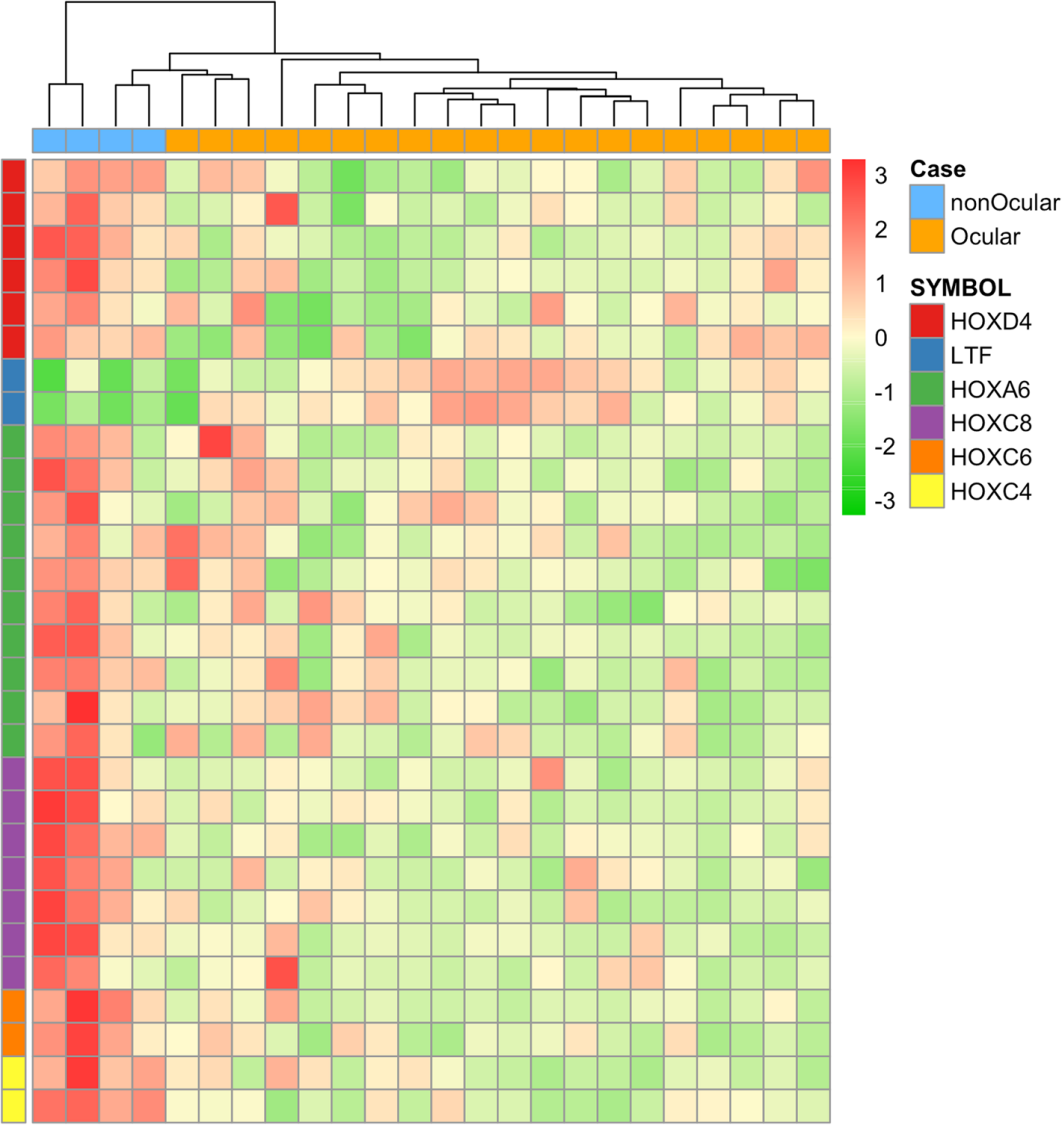
Global methylation profiling of orbitofacial and non-orbitofacial neurofibromas. Centered beta values for probes of differentially methylated genes (*HOXC8, HOXC4, HOXC6, HOXA6, HOXD4 and LTF*). HOX transcription factors were found hypomethylated in the orbitofacial neurofibromas compared with the non-orbital. Conversely, LTF was relatively hypermethylated in orbitofacial NF

**Fig. 3:**
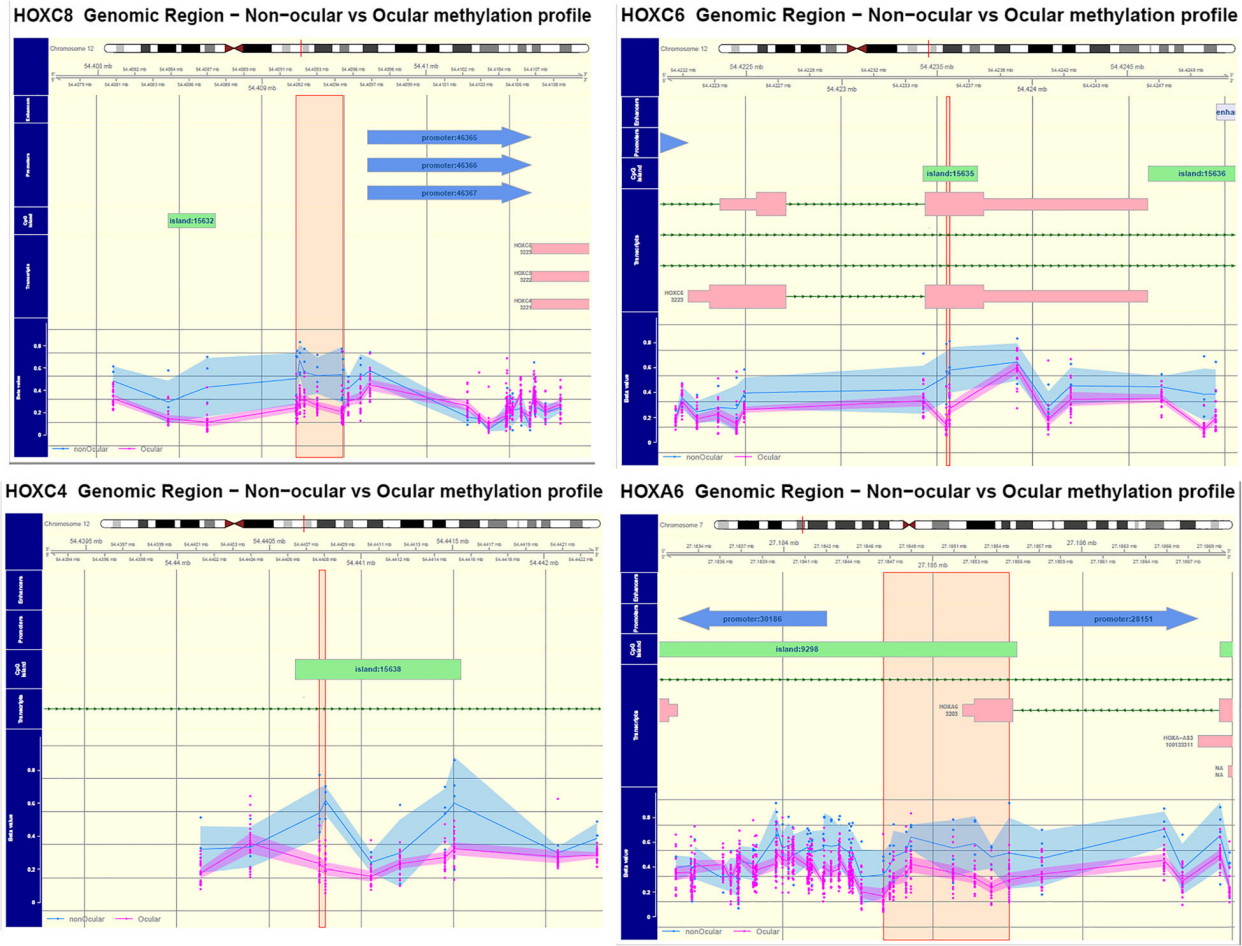
*HOX* family transcription factors are hypomethylated in orbitofacial neurofibromas. Genomic maps demonstrating promoter hypomethylation of *HOXC8, HOXC6, HOXC4* and *HOXA6* in orbitofacial neurofibromas. The x-axis corresponds to the chromosome location of individual HOX genes differentially methylated regions (DMR) in or near the promoter in orbitofacial NF (pink dots and plot) vs non-orbitofacial NF (blue dots and plot). The y-axis demonstrates the methylation status near the gene of interest as Beta values. Beta values closest to 0 denote decreased methylation, and values closest to 1 increased methylation. HOX transcription factor genes are relatively hypomethylated in orbitofacial NF compared to non-orbitofacial NF

Differential methylation analysis identified 7 differential methylated regions between the orbitofacial (*n* = 20) and non-orbitofacial (*n* = 4) neurofibromas (FWER ≤0.1; *P* ≤ 0.0001) (Fig. 2 and Table 1). These DMRs were found predominantly within or next to the *HOX* family of transcription factors genes. *HOXC8, HOXC4, HOXC6, HOXA6 and HOXD4* were found hypomethylated in the orbitofacial NF compared with the non-ocular (Fig. 3). Conversely, the transferrin family member *LTF* was relatively hypermethylated in orbitofacial NF compared to the non-orbital neurofibromas (Fig. 2). Gene methylation differences were further adjusted by age (Supplementary table 1).

**Table 1:** Differentially methylated genes in orbitofacial vs. non-orbitofacial neurofibromas

Chromosome	Start	End	Effect Size	p-value	FWER	Nearest Gene
chr12	54,409,207	54,409,491	−0.2798	3.77E-07	0.015	HOXC8
chr12	54,440,773	54,440,806	−0.36069	7.54E-07	0.03	HOXC4
chr7	27,184,667	27,185,732	−0.19761	1.26E-06	0.045	HOXA6
chr2	1.77E+ 08	1.77E+ 08	−0.2555	1.13E-06	0.045	HOXD4
chr12	54,423,549	54,423,566	−0.34654	2.01E-06	0.065	HOXC6
chr3	46,484,250	46,484,379	0.333892	2.64E-06	0.085	LTF

### HOXC8 protein levels are higher in orbitofacial compared to non-orbital neurofibromas

**Fig. 4:**
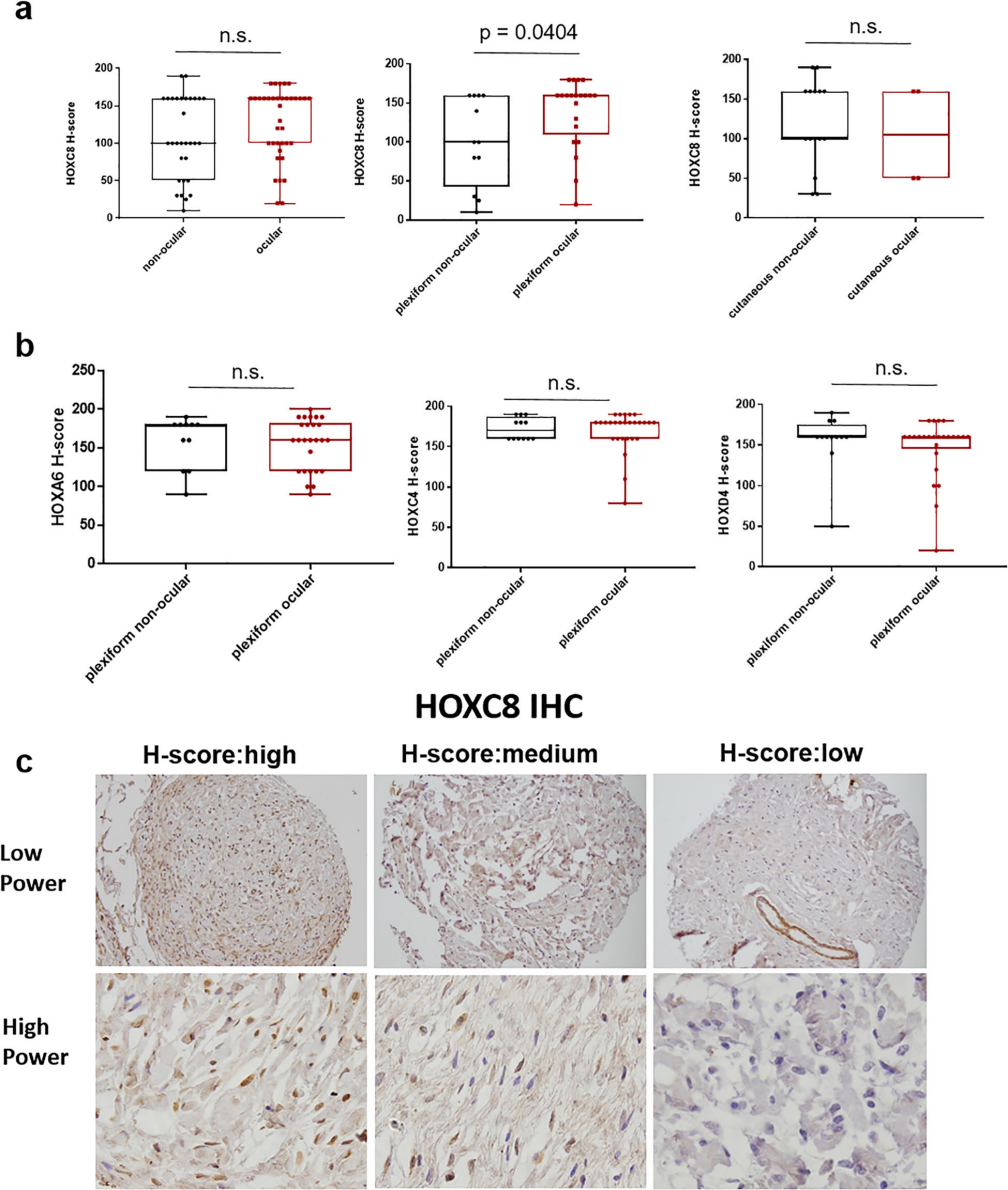
HOXC8 protein is increased in plexiform orbitofacial neurofibromas compared with plexiform neurofibromas at other sites. **a** Immunohistochemical H-score for HOXC8 was increased in plexiform orbitofacial compared to plexiform non-orbitofacial. No statistical significant differences were seen in non-orbitofacial vs orbitofacial as a whole or cutaneous non-orbitofacial vs cutaneous orbitofacial (unpaired T-test with Welch’s correction). **b** No differences were noted between plexiform non-orbitofacial vs. plexiform orbitofacial immunoreactivity for HOXA6, HOXC4, and HOXD4 (unpaired T-test with Welch’s correction). **c** Representative images for H-score method assigned a score of 0–300 to each patient. The patient samples were classed as low (H-score 0–100), medium (100–200), and high (200–300). Forty-six orbitofacial and 31 non-orbitofacial neurofibromas were analyzed

Immunohistochemistry of the 46 orbitofacial NFs and 31 non-orbital NFs showed no significant differences in protein expression between the two groups, although HOXC8 protein levels were higher in orbitofacial plexiform NFs (average H-score 126, range 0–180) compared to non-orbital plexiform NFs (average H score 100, range 10–160) (*P* = 0.04) (Fig. 4). We found no significant differences in protein expression between the orbitofacial NFs vs. non-orbital groups for HOXC4 (average H-score 170, range 80–190, vs. average H-score 172, range 160–190), HOXA6 (average H-score 155, range 90–190 vs. average H-score 158, range 90–190), or HOXD4 (average H-score 146, range 20–180 vs average H-score 155, range 50–190) (*p* > 0.05). Clinicopathologic features of the cohort are summarized in Supplementary Table 2. Next, we studied the ki67 proliferation index using immunohistochemistry. Proliferation index was slightly higher on average in the orbitofacial NFs compared with the non-orbital NFs (Supplementary Figure 1).

### HOXC8 mRNA expression is increased in orbitofacial compared to non-orbital neurofibromas

**Fig. 5:**
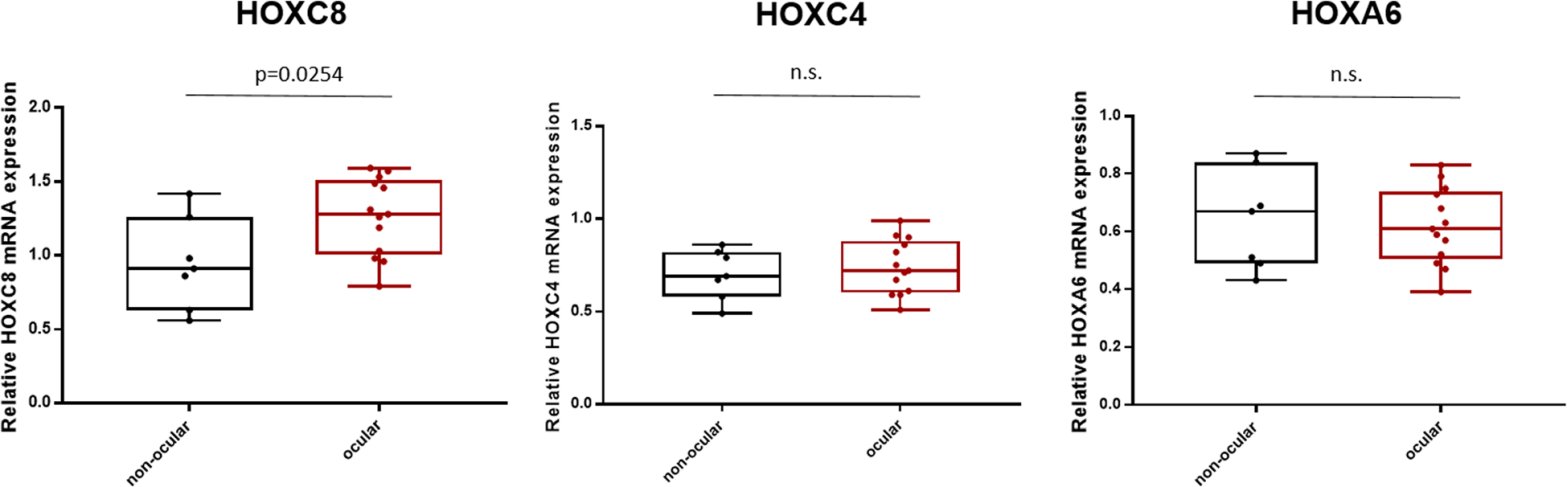
*HOXC8* mRNA expression was significant higher in orbitofacial compared to non-orbitofacial neurofibromas. qPCR analysis confirmed a significant higher mRNA level in orbitofacial samples compared to non-orbitofacial NF (*P = 0.0254). No significant difference was detected for *HOXC4* and *HOXA6* (unpaired T-test with Welch’s correction)

Next we studied mRNA expression levels in orbitofacial NF (*n* = 13) samples compared to non-orbital neurofibromas (*n* = 7) using qRT-PCR. *HOXC8* mRNA levels were ~ 1.3 fold higher in orbitofacial neurofibromas compared to non-orbitofacial neurofibromas (*P* = 0.0254) which was in line with the higher protein expression (Fig. 5). No significant differences were detected for mRNA levels in *HOXC4* and *HOXA6.* Clinicopathologic features of the cases studied are summarized in Supplementary table 2.

### *HOXC8* increases human Schwann cell growth

**Fig. 6:**
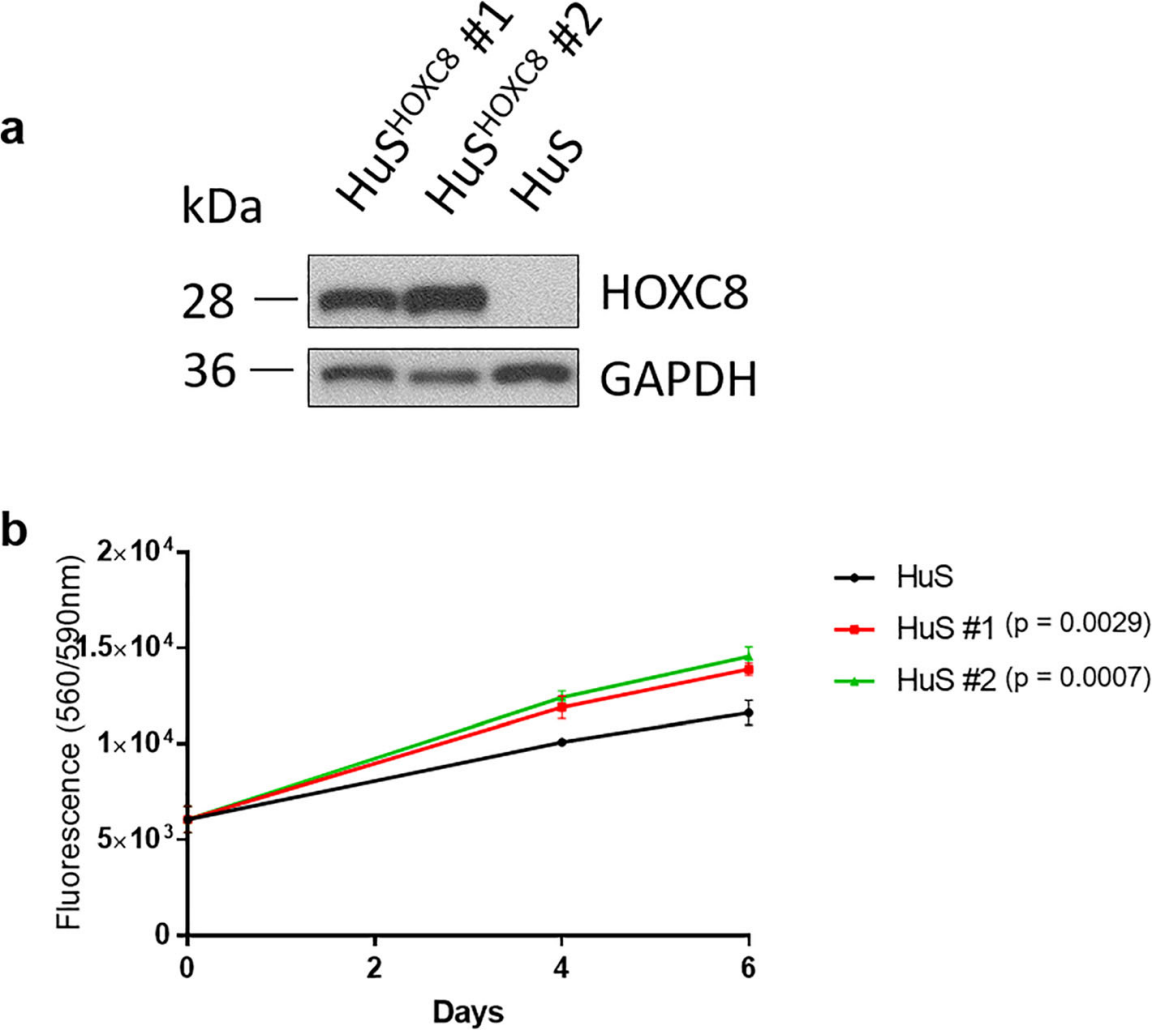
Overexpression of HOXC8 leads to an increase in human Schwann cell growth. **a** Overexpression of *HOXC8* was independently confirmed in 2 Schwann cell clones via Western Blot assay compared to the donor human Schwann cell line. **b** After selection, both *HOXC8* huSchwann cell lines showed increased growth compared with the donor huSchwann cell line measured with cell titer blue assay (**P = 0.0029; ***P = 0.0007 by one-way ANOVA with post-hoc Tukey multiple comparisons test)

We wanted to study the effect of overexpression of HOXC8 in a human Schwann cell line. For this we overexpressed *HOXC8* through viral transduction. After selection, both *HOXC8* human Schwann cell clones showed increased protein expression compared to the donor human Schwann cell line (Fig. 6a). To investigate if higher *HOXC8* protein will have an effect on cell growth, we performed cell titer blue experiments over 6 days. Both clones of the *HOXC8* overexpressing cell lines showed significant increase in cell viability compared with the donor human Schwann cell line (clone#1 *P* = 0.0029; clone#2 *P* = 0.0007) (Fig. 6b).

## Discussion

The importance of epigenetic changes has been increasingly recognized in a variety of physiologic and pathologic states, particularly neoplasms. In the current study, we identify important epigenetic differences between orbitofacial NF and those occurring at other sites, which may partially explain the increased aggressiveness of orbitofacial NFs. In particular, *HOX* family genes appear to be altered by differential methylation in NFs.

The *HOX* family encompass a group of genes arranged into four distinct clusters (HOXA, HOXB, HOXC, HOXD) in various chromosomal locations [26]. They are known to play a critical role during development, encoding proteins that provide critical positional information during morphogenesis through variable protein composition, leading to regional patterning [8]. The *HOX* genes are sequentially expressed during development, underlying the embryologic process known as the ‘temporal collinearity’ of the ‘HOX clock’ [9, 17].

Recent evidence has supported a role for *HOX* genes in physiologic and pathologic states involving stem cell biology, including cell hierarchy, positional identity, self-renewal and differentiation [22]. Altered expression and mutations leading to genetic predisposition have been studied in a variety of cancer types, including gynecologic and hematologic malignancies [26]. In our study, we found decreased DNA methylation in several *HOX* family genes in orbitofacial NFs. In particular, *HOXC8* demonstrated increased mRNA and protein expression in orbitofacial NFs compared to NFs arising at other sites.

Altered HOXC8 levels have been documented in a variety of tumor types, and have been shown to be of biological and prognostic significance. For instance, high expression levels of HOXC8 and HOXC6 by immunohistochemistry were associated with poorer prognosis in a cohort of patients with esophageal squamous cell carcinoma [10], and increased gene expression was associated with worse outcome in cervical carcinoma [16]. In another study, HOXC8 levels were increased in non-small cell lung cancer and was correlated with lymph node metastasis, differences that were thought to be mediated by TGFβ1 [27]. HOXC8 has also been found to regulate proliferation, migration, and epithelial mesenchymal transition in triple negative breast cancer [12] and invasion in prostate cancer [3]. However, a possible tumor suppressor role has also been reported in other studies, where overexpression of *HOXC9* leads to decreased growth of nasopharyngeal carcinoma and stem cell properties in breast cancer cells [41]. Also, *HOXC8* expression has been shown to be inversely correlated with metastasis in pancreatic cancer [1].

The regulation of *HOX* genes during development and disease is complex, but epigenetic changes such as DNA methylation appear to be important mediators [44]. For example, *HOXC8* methylation seems to regulate cashmere fiber length in the cashmere goat [4]. Variable *HOX* gene methylation has also been described in oral squamous cell carcinoma cell lines [46].

The results from our study suggest that *HOX* genes may play a role in the development of various NF subtypes. Of relevance, in a recent study Chen et al. demonstrated that inactivation of *Nf1* in mice in a *HOXB7* expressing neural crest lineage cell leads to the development of cutaneous and plexiform NFs in restricted anatomic distributions [7].

*LTF* (lactoferrin) is a member of the transferrin gene family. It is an iron binding protein which participates in the reduction of oxidative stress and inflammation [25]. In our study it was the only significant gene to be differentially hypermethylated in orbitofacial NF. *LTF* has been found to be down-regulated in papillary thyroid cancer [14], gastric cancer [28], and underexpressed in association with hypermethylation in prostate cancer [36, 42] and nasopharyngeal carcinoma [47], supporting a role as a tumor suppressor gene in a variety of cancer types.

One of the caveats of our study is that clinical data, including precise age of onset, time of progression, extent of invasion as well as the precise *NF1* mutations associated with their development were not studied. In addition, current efforts to more precisely characterize cutaneous neurofibromas, integrating clinical and pathological data are ongoing [34], and will have management implications. Orbitofacial neurofibromas are relatively rare, and larger cohorts will require multiinstitutional studies, with prospective clinical data that includes age of onset, precise clinicopathologic subtypes and *NF1* mutations at a minimum, allowing to adjust for all these variables. Future studies into the molecular characterization of orbitofacial NF, and the relevance of *HOX* genes, will certainly include these variables, given their relevance to the study of NF1-associated neoplasia.

In summary, we report that there is differential methylation and expression of *HOX* genes in orbitofacial NFs when compared to NFs developing at other sites. Overexpression of *HOXC8* leads to increased cell growth in non-neoplastic Schwann cells, suggesting a possible contribution to the increased aggressiveness in certain subtypes of NF. These findings warrant further investigation in clinical cohorts and animal models to continue the characterization of *HOX* genes and their role in the pathobiology of NFs, and develop rational therapies for tumors that although benign, are associated with disproportionate morbidity with current treatment regimens.

## Supplementary information

**Additional file 1 Table S1.** Differentiatially methylated probes and corresponding genes in orbitofacial vs. non-orbitofacial neurofibromas adjusted by age.

**Additional file 2 Table S2.** Clinicopathologic data of orbitofacial and non-orbitofacial neurofibromas.

**Additional file 3.**

## Data Availability

The datasets generated and/or analysed during the current study are available in the GEO repository.
