# Correction to: Differential gene methylation and expression of HOX transcription factor family in orbitofacial neurofibroma

**DOI:** 10.1186/s40478-020-00944-3

**Published:** 2020-05-14

**Authors:** Antje Arnold, Eddie Luidy Imada, M. Lisa Zhang, Deepak P. Edward, Luigi Marchionni, Fausto J. Rodriguez

**Affiliations:** 1 Departments of Pathology, Johns Hopkins University School of Medicine, Baltimore, MD United States; 2 Departments of Ophthalmology and Sidney Kimmel Comprehensive Cancer Center, Johns Hopkins University School of Medicine, Baltimore, Maryland (MD) USA; 3 Department of Pathology, Massachusetts General Hospital, Boston, MA USA; 4 King Khaled Eye Specialist Hospital, Riyadh, Saudi Arabia; 5 Department of Ophthalmology and Visual Sciences, University of Illinois College of Medicine, Chicago, IL USA; 6 Johns Hopkins University School of Medicine, Sheikh Zayed Tower, Room M2101, 1800 Orleans Street, Baltimore, MD 21231 USA

**Correction to: Acta Neuropathol Commun 2020 8: 62**

**https://doi.org/10.1186/s40478-020-00940-7**

Following publication of the original article [1], the authors identified an error in the author name of Lisa Zhang,

- The incorrect author name is: Lisa Zhang
- The correct author name is: M. Lisa Zhang

The author group has been updated above and the original article [1] has been corrected.
