# Supplementary figures and images for: Differential gene methylation and expression of HOX transcription factor family in orbitofacial neurofibroma
Source: Acta Neuropathol Commun. 2020 May 4;8:62. doi: 10.1186/s40478-020-00940-7 (PMC7197183; doi:10.1186/s40478-020-00940-7)

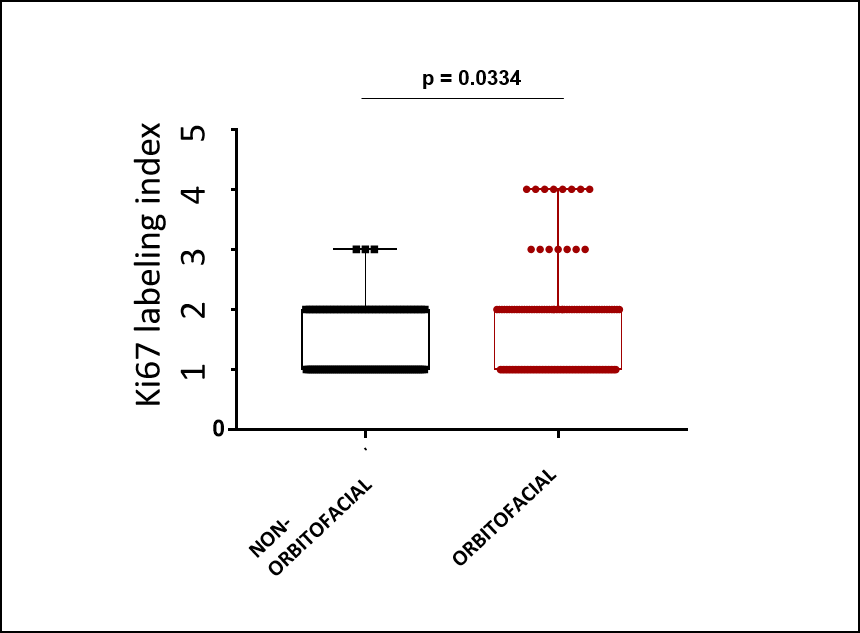

Supplement: Supplementary file 3 — Additional file 3. [file 40478_2020_940_MOESM3_ESM.tif]
